# The Association of Pulmonary Tuberculosis, Abnormal Glucose Tolerance, and Type 2 Diabetes Mellitus: A Hospital-Based Cross-Sectional Study

**DOI:** 10.7759/cureus.19758

**Published:** 2021-11-19

**Authors:** Jayashankar CA, Manjunath BM, Venkata BharatKumar Pinnelli, Venkataramana Kandi, Shalini AS, Harsha A Mathew, Honika Gundreddy, Fareeha Afreen, Sabitha Vadakedath

**Affiliations:** 1 General Medicine, Vydehi Institute of Medical Sciences and Research Centre, Bengaluru, IND; 2 Biochemistry, Vydehi Institute of Medical Sciences and Research Centre, Bengaluru, IND; 3 Clinical Microbiology, Prathima Institute of Medical Sciences, Karimnagar, IND; 4 General Medicine, Pushpagiri Institute of Medical Sciences, Thiruvalla, IND; 5 Biochemistry, Prathima Institute of Medical Sciences, Karimnagar, IND

**Keywords:** glycated hemoglobin (hba1c), impaired glucose tolerance, glycated hemoglobin, oral glucose tolerance test, diabetes mellitus, tuberculosis

## Abstract

Introduction

Tuberculosis (TB) is an infectious disease caused by *Mycobacterium tuberculosis* and is the second leading infectious cause of death worldwide. The higher prevalence of pulmonary TB in patients with type 2 diabetes mellitus (T2DM) is a well-known fact. The inverse relationship is also being increasingly recognized. Very few studies are available on the correlation of glycemic parameters with grades of sputum acid-fast bacilli (AFB) positivity and disease severity. Hence, this study is undertaken to determine the prevalence of impaired glucose tolerance (IGT), new-onset T2DM, and to correlate glycemic parameters with sputum positivity grades in pulmonary TB patients.

Methods

The is a cross-sectional study that included 93 patients with confirmed pulmonary TB, who presented to the General Medicine and Pulmonary Medicine departments of a tertiary care teaching hospital in southern India. All the patients included in the study underwent oral glucose tolerance (OGTT; 75 g) and glycated hemoglobin (HbA1c) tests. The results were analyzed and interpreted using statistical applications (SPSS software version 21, IBM Corp., Armonk, NY).

Results

Among the 93 patients included in the study, 73 (78.4%) were males and the mean age was 42.5+1.5 years. The OGTT revealed abnormal results in 44 (47.3%) patients. Thirteen (14%) patients showed IGT and 31 (33.3%) had newly been detected with T2DM. The mean HbA1C of the study participants was noted to be 6.413%.

Conclusion

The prevalence of IGT and T2DM among pulmonary TB patients was noted to be 14% and 33.3%, respectively. The grade of sputum positivity and the severity of the disease did not correlate with the serum of HbA1c levels.

## Introduction

Tuberculosis (TB) is an infectious disease caused by *Mycobacterium tuberculosis* and is the second leading infectious cause of death worldwide. The World Health Organization (WHO) declared TB as a global health emergency in 1996 [1]. TB is a major public health problem in India, and it accounts for one-fifth of the global TB incident cases [2]. The global TB report 2019, released by the WHO indicated that there were 10 million new cases and 1.5 million deaths reported in the year 2018 [3]. It is estimated that more than two million people suffer from active TB, and around 330,000 Indians die every year due to TB [4,5].

The higher prevalence of impaired glucose tolerance (IGT) and type 2 diabetes mellitus (T2DM) in the TB population is being increasingly identified both in India and elsewhere in the world. Also, it has been perceived that the combination of T2DM and TB may interfere with the therapeutic intervention and influence the disease course. Therefore, screening TB patients undergoing therapy for glycemic status assumes increased significance [6]. TB patients with T2DM were found predisposed to infection relapses, treatment failures, increased mortality, delayed mycobacterial clearance as observed by previous studies [7-9]. The IGT may occur transiently during pulmonary TB owing to the stress of prevailing infection and it has been shown to improve following effective anti-tubercular therapy [10]. Previous studies have shown a high prevalence of abnormal glucose tolerance (GT) in TB patients with rates ranging from 2% to 41% after OGTT [11]. The relationship between pulmonary TB and the development of abnormal GT is not well documented. Also, the data on the prevalence of T2DM and IGT among pulmonary TB patients are limited. Very few studies are available on the correlation of glycemic parameters with grades of sputum acid-fast bacilli (AFB) positivity and disease severity. Therefore, this study is undertaken to determine the prevalence of new-onset IGT and T2DM in pulmonary TB patients and correlate the glycemic parameters with the grades of sputum AFB positivity and disease severity.

## Materials and methods

This is a cross-sectional study done on pulmonary TB patients who were attending the outpatient departments of General Medicine and Pulmonary Medicine attached to a tertiary care teaching hospital in southern India. The study was conducted between January 2018 and June 2019 and was approved by the ethics committee of the institution, Vydehi Institute of Medical Sciences and Research Centre (EC Reg No: ECR/747/Inst/KA/2015). Informed consent was obtained from all the study participants.

Inclusion and exclusion criteria

Patients presenting with fever, cough, and expectoration for more than two weeks duration, and those who gave a detailed history were recruited into the study after a thorough clinical examination. The early morning sputum samples were collected from each patient and sent to the microbiological lab for the presence of AFB on two consecutive days. All other necessary and supportive investigations like complete blood count (CBC), erythrocyte sedimentation rate (ESR), and posterior/anterior view chest X-ray were done.

Patients with prior human immunodeficiency virus (HIV) infection, T2DM, thyroid disease, adrenal disorders, other critical illnesses, malignancy, pregnant and lactating women, and patients on drugs like oral contraceptive pills, glucocorticoids, diuretics, and statins were excluded from the study.

A fasting blood sample was collected from all the study participants for the estimation of fasting plasma glucose (FPG) and glycated hemoglobin (HbA1c). Each study participant also underwent a 75 g oral glucose tolerance test (OGTT) and consumed 75 g anhydrous glucose in 250-300 mL of water. Blood was drawn to measure postprandial glucose (PPG) at one and two-hour intervals after consumption of the oral glucose load. If the patient vomited, testing was stopped and repeated on another day. In accordance with WHO criteria, two-hour PPG ≥200 mg/dL was considered diagnostic of diabetes mellitus, and two-hour PPG 140-199 mg/dL was considered diagnostic of IGT.

Statistical analysis

The quantitative data were represented as mean and standard deviation (SD). Categorical and nominal data were expressed in percentage. The t-test was used to analyze quantitative data and nonparametric data were analyzed by Mann Whitney test. The categorical data were analyzed by using the chi-square test. The significance threshold of the p-value was set at <0.05. All analyses were conducted by using SPSS software version 21 (IBM Corp., Armonk, NY).

## Results

In all, 93 patients with pulmonary TB were included in the study after satisfying the inclusion and exclusion criteria. The mean age of patients was 42±15 years, and 73 (78.4%) of patients were male.

Sputum was graded as scanty in 11.8% cases while grade 1+, 2+, 3+ was recorded in 21.5%, 29% and 37.6% cases, respectively. The distribution of pulmonary TB patients depending on the grade of sputum positivity and abnormal glucose results after GTT are shown in Tables 1 and 2, respectively.

**Table 1:** Distribution of study cases as per sputum grade Scanty: 1-9 acid-fast bacilli in 100 oil immersion fields; 1+: 10-99 acid-fast bacilli in 100 oil immersion fields; 2+: 1-10 acid-fast bacilli in each oil immersion field; 3+: >10 acid-fast bacilli in each oil immersion field (grading was based on the Revised National Tuberculosis Program, India guidelines).

Sputum grade	N	%
Scanty	11	11.8%
1+	20	21.5%
2+	27	29.0%
3+	35	37.6%
Total	93	100.0%

**Table 2:** Distribution of study cases as per the results of oral glucose tolerance test OGTT: oral glucose tolerance test; IGT: impaired glucose tolerance; DM: diabetes mellitus

OGTT	N	%
Normal	49	52.7%
IGT	13	14.0%
DM	31	33.3%
Total	93	100.0%

On OGTT, 44 patients (47.3%, 95% CI: 36.7-59.3%) had abnormal results. Out of the total 44 cases with abnormal results, 14% had IGT and 33.3% had T2DM. There was no significant association between sputum AFB grade and diagnosis by OGTT as shown in Table 3.

**Table 3:** Comparison of oral glucose tolerance test results with sputum acid-fast bacilli grade AFB: acid-fast bacilli; DM: diabetes mellitus; IGT: impaired glucose tolerance; Chi-square (χ^2^)=5.823, degrees of freedom (df)=6, p=0.443.

		Diagnosis							
		DM		IGT		Normal		Total	
		N	%	N	%	N	%	N	%
Sputum AFB Grade	Scanty	6	54.5%	1	9.1%	4	36.4%	11	100.0%
	1+	5	25.0%	1	5.0%	14	70.0%	20	100.0%
	2+	8	29.6%	5	18.5%	14	51.9%	27	100.0%
	3+	12	34.3%	6	17.1%	17	48.6%	35	100.0%
	Total	31	33.3%	13	14.0%	49	52.7%	93	100.0%

There was no significant difference in the mean fasting, one hour, and two hours plasma glucose values after OGTT with respect to the grade of sputum positivity as shown in Table 4.

**Table 4:** Mean comparison of blood sugar levels for oral glucose tolerance test among various sputum grades OGTT: oral glucose tolerance test; SD: standard deviation; SE: standard error; analysis of variance (ANOVA) was performed and a p-value <0.05 is considered as significant.

Grade of sputum		N	Mean	SD	SE	95% Confidence interval for mean		Minimum	Maximum	p-value
						Lower bound	Upper bound			
OGTT fasting	Scanty	11	128.18	21.826	6.581	113.52	142.84	98	178	0.351
	1+	20	120.20	24.859	5.559	108.57	131.83	72	201	
	2+	27	128.52	26.684	5.135	117.96	139.07	92	186	
	3+	35	132.77	23.650	3.997	124.65	140.90	100	202	
	Total	93	128.29	24.682	2.559	123.21	133.37	72	202	
OGTT at 1 hour	Scanty	11	166.45	23.304	7.026	150.80	182.11	128	200	0.600
	1+	20	161.60	24.221	5.416	150.26	172.94	116	225	
	2+	27	225.37	327.956	63.115	95.64	355.11	111	1858	
	3+	35	177.86	33.774	5.709	166.26	189.46	126	260	
	Total	93	186.81	177.911	18.448	150.17	223.45	111	1858	
OGTT at 2 hours	Scanty	11	151.82	24.024	7.244	135.68	167.96	128	192	0.336
	1+	20	138.70	21.767	4.867	128.51	148.89	110	210	
	2+	27	144.15	24.323	4.681	134.53	153.77	110	200	
	3+	35	150.69	29.316	4.955	140.62	160.76	120	240	
	Total	93	146.34	25.890	2.685	141.01	151.68	110	240	

The study results revealed no statistically significant difference in mean HbA1c with respect to the grade of sputum positivity as shown in Table 5.

**Table 5:** Mean comparison of glycated hemoglobin among various sputum grades HBA1c: glycated hemoglobin; SD: standard deviation; SE: standard error; p-value <0.05 is considered as significant.

HbA1c									p-value
	N	Mean	SD	SE	95% confidence interval for mean		Minimum	Maximum	
					Lower bound	Upper bound			
Scanty	11	6.418	0.9020	0.2720	5.812	7.024	4.8	7.8	0.575
1+	20	6.290	1.2226	0.2734	5.718	6.862	4.8	9.2	
2+	27	6.293	1.1422	0.2198	5.841	6.744	4.2	8.6	
3+	35	6.651	1.1587	0.1959	6.253	7.049	5.2	9.2	
Total	93	6.442	1.1359	0.1178	6.208	6.676	4.2	9.2	

## Discussion

Tuberculosis is widely prevalent in developing countries including India. Also, the burden of T2DM is increasing throughout the world. The available literature suggests that there is an increased incidence of tuberculosis among T2DM patients. However, there are conflicting views of the association of impaired glucose tolerance and diabetes with TB.

It is unclear if glucose intolerance and diabetes mellitus among TB patients were coincidental or a sign of true association. Nevertheless, there is a perception that this relationship between TB and impaired glucose tolerance and T2DM may be a result of prevalent diabetes mellitus which was being newly diagnosed in patients receiving expanded medical services related to TB treatment.

There are various mechanisms by which tuberculosis can cause impaired glucose tolerance. Since TB is a chronic inflammatory condition, it can cause the release of stress hormones such as cortisol which raises blood sugar levels. The release of other cytokines, chemokines, and the accumulation of tubercular proteins in the pancreas may cause pancreatic dysfunction, thereby destroying the pancreatic tissue [12,13]. This causes low production of insulin and results in transient glucotoxicity.

The association of diabetes and transient hyperglycemia/impaired glucose tolerance among TB patients is often unrecognized [13]. In 1995, Jawad et al. followed up 106 pulmonary TB patients (63 were males and 43 females with a mean age of 39.3 years). OGTT showed glucose intolerance in 52(49%) patients. Among them, 31 revealed IGT, and 21 showed diabetes mellitus. When the OGTT was repeated in 23 cases after adequate antitubercular therapy, 13 (56.5%) patients reverted to normal glucose tolerance indicating that the glucose intolerance observed during active pulmonary TB improves or normalizes after adequate therapy [11]. Similar conclusions were obtained in many other cross-sectional studies conducted across the globe that reemphasizes the need to screen and regularly assess all TB patients for impaired glucose tolerance [6,14-19].

A recent study by Gautam et al. had performed a meta-analysis of observational studies in the year 2020. This study observed that a high burden of diabetes among TB patients was noted in South Asia. Also, it was noted that when compared to non-diabetic TB patients, patients with TB and diabetes were associated with higher odds of mortality and treatment failure but were not associated with multi-drug resistant TB [20].

The significance of reinforcing the need to screen for glycemic status and achieve good glycemic control among TB patients for better treatment outcomes was suggested by previous studies [8,21-24]. Because diabetes is a common non-communicable disease, it is important to adequately investigate the association of diabetes and impaired glucose tolerance among patients with TB.

However, a study conducted by Pizzol et al. from Mozambique in the year 2016 observed a low prevalence of T2DM in newly diagnosed pulmonary TB patients [25]. The results of this study point to the potential role played by antitubercular therapy in the development of impaired glucose tolerance and diabetes among TB patients that require further investigations.

In the present study, no significant association was observed between deranged OGTT values and higher grades of sputum positivity (p=0.443). A total of 63.6% of cases with scanty reports had impaired values as compared to 51.4% in cases with grade 3+ sputum. Mean fasting and blood sugar levels at one hour were higher in cases with grade +2/+3 sputum positivity as compared to scanty/+1 grade. However, there was no significant difference in mean fasting, one-hour, and two-hour blood glucose values with respect to grade of sputum positivity and disease severity.

HbA1c was not significantly associated with the grade of sputum positivity and disease severity in the present study. HbA1c levels were 6.418% in cases with less severe disease (sputum grade=scanty), whereas 6.290%, 6.293% and 6.651% in case of grade +1, +2, and +3, respectively (p=0.575).

Study limitations

The limitation of the study is that it is done amongst a small group of people confined to a particular geographical area/region, and extrapulmonary TB patients were excluded. Since this is a cross-sectional study, no cases were followed up to understand the glycemic and related outcomes after anti-TB treatment.

## Conclusions

In the present study, a high prevalence of IGT and T2DM was observed among TB patients. Association between T2DM and TB is the next challenge for global TB management. Improved understanding of the bidirectional relationship of the two diseases is necessary for proper planning and collaboration to reduce the dual burden. In people with TB, it may be appropriate to actively screen for T2DM. Prevention, screening, and treatment of both diseases appear more effective and perhaps a model similar to the TB-HIV program may be the best approach. The scarcity of data on the association of T2DM with TB and the need for consensus on the improved screening methods and the timing of screening for impaired glucose tolerance in TB patients require further large-scale follow-up studies.
